# Pathogenicity of West Nile Virus Lineage 1 to German Poultry

**DOI:** 10.3390/vaccines8030507

**Published:** 2020-09-05

**Authors:** Cora M. Holicki, Friederike Michel, Ana Vasić, Christine Fast, Martin Eiden, Cristian Răileanu, Helge Kampen, Doreen Werner, Martin H. Groschup, Ute Ziegler

**Affiliations:** 1 Institute of Novel and Emerging Infectious Diseases, Federal Research Institute for Animal Health, Friedrich-Loeffler-Institut, 17493 Greifswald-Insel Riems, Germany; cora.holicki@fli.de (C.M.H.); friederike.michel@gmx.net (F.M.); christine.fast@fli.de (C.F.); martin.eiden@fli.de (M.E.); martin.groschup@fli.de (M.H.G.); 2 Institute of Infectology, Federal Research Institute for Animal Health, Friedrich-Loeffler-Institut, 17493 Greifswald-Insel Riems, Germany; anavasicdvm@gmail.com (A.V.); cristian.raileanu@fli.de (C.R.); helge.kampen@fli.de (H.K.); 3 Biodiversity of Aquatic and Semiaquatic Landscape Features, Leibniz-Centre for Agricultural Landscape Research, 15374 Muencheberg, Germany; doreen.werner@zalf.de

**Keywords:** West Nile virus, arbovirus, experimental infection, pathogenesis, poultry, chickens, ducks, geese, sentinel, *Culex pipiens*

## Abstract

West Nile virus (WNV) is a mosquito-borne virus that originates from Africa and at present causes neurological disease in birds, horses, and humans all around the globe. As West Nile fever is an important zoonosis, the role of free-ranging domestic poultry as a source of infection for humans should be evaluated. This study examined the pathogenicity of an Italian WNV lineage 1 strain for domestic poultry (chickens, ducks, and geese) held in Germany. All three species were subcutaneously injected with WNV, and the most susceptible species was also inoculated via mosquito bite. All species developed various degrees of viremia, viral shedding (oropharyngeal and cloacal), virus accumulation, and pathomorphological lesions. Geese were most susceptible, displaying the highest viremia levels. The tested waterfowl, geese, and especially ducks proved to be ideal sentinel species for WNV due to their high antibody levels and relatively low blood viral loads. None of the three poultry species can function as a reservoir/amplifying host for WNV, as their viremia levels most likely do not suffice to infect feeding mosquitoes. Due to the recent appearance of WNV in Germany, future pathogenicity studies should also include local virus strains.

## 1. Introduction

West Nile virus (WNV; *Flaviviridae*, *Flavivirus*) is a widely-dispersed zoonotic arbovirus (arthropod-borne virus) and causative agent of severe neurological disease in humans and animals. As an ecological generalist, WNV can be transmitted by various mosquito species and it is capable of infecting a diverse range of vertebrate hosts, such as mammals, amphibians, and reptiles [1]. Birds, however, are natural reservoirs with numerous species that are capable of producing viremia levels sufficient to infect mosquitoes (primarily *Culex* species) and thereby perpetuate the transmission cycle. Passeriformes, such as house sparrows (*Passer domesticus*), Falconiformes, and Strigiformes, as, for example, great grey owls (*Strix nebulosa*), have demonstrated a high susceptibility to WNV, developing severe neurological signs. Other vertebrate hosts, such as humans and horses, are considered to be incidental or dead-end hosts. They become infected via bridge vectors, which are mosquito species with an indiscriminate host selection, feeding on avian as well as mammalian species. Horses and humans usually seroconvert without clinical symptoms; however, in rare cases they can develop a life-threatening neuroinvasive disease. Their viremia levels are too low to pass on the virus to mosquitoes [2].

WNV has its origin in the West Nile District of Uganda, where it was first isolated from the blood of a febrile patient in 1937 [3]. Until the early 1990s, the virus was not perceived as a serious threat, as birds were considered resistant [4,5] and human infections were only associated with sporadic outbreaks in Africa, the Near and Middle East, Asia, and Europe. In Europe, the epidemiology and transmission dynamics of WNV changed in the mid-1990s with increasing disease severity and frequency among humans and horses [6]. This trend was significantly reinforced by the appearance of WNV lineage 2 in 2004 in Hungary [7], with a consequent rise in the number of neuroinvasive disease cases in humans. Countries that were most affected were Romania (e.g., in 1996 [8] and 2010 [9]), Russia (e.g., in 1999 [10]), France (e.g., in 2000 [11]), Greece (e.g., in 2010 [12]), Italy (e.g., in 2011 [13,14,15]), and Serbia (e.g., in 2012 [16]). At the same time, WNV became highly pathogenic for the avifauna with increasing morbidity and mortality rates amongst free-ranging bird species [17]. This became particularly apparent in 1999 when WNV strain NY99-flamingo382-99 (GenBank accession no. AF196835) emerged in the western hemisphere in New York City [18,19]. WNV quickly managed to establish itself throughout the United States of America (USA) due to the presence of immunologically naïve bird populations and the putatively high virulence of this virus strain [20,21]. The virus was eventually found in 65 mosquito species and at least 326 avian species [22]. Free-ranging birds, such as the American crow (*Corvus brachyrhynchos*), blue jay (*Cyanocitta cristata*), and American robin (*Turdus migratorius*), were most affected and their population sizes declined noticeably with the arrival of WNV in the USA [23]. 

At present, WNV activity is also reappearing yearly in Europe with recurring outbreaks among humans and horses in the south and southeast. Especially, the years 2018 and 2019, with exceptionally long-lasting warm and dry summers, provided favorable climatic conditions for WNV transmission and spread in Europe [24]. This resulted not only in an early start of the WNV transmission season, but also let the number of human and equine cases exceed the total of the previous seven years. More than 2,000 human WNV infections were diagnosed in 2018, of which 180 were fatal [25]. That year was also noteworthy with regard to WNV expansion in Europe with more countries affected than in preceding transmission seasons [26]. 

Infection experiments with local avian species are needed to better understand the eco-epidemiology of WNV in Europe. Similar studies have already been carried out using North American (Neartic) bird species and the NY99 WNV strain (for review see Pérez-Ramírez et al., 2014 [27]), but the susceptibility of Palearctic as compared to Nearctic bird species is possibly different as well as the pathogenicity of circulating strains [27]. The role of WNV for domestic poultry, such as chickens, ducks, and geese, is also not fully understood. WNV outbreaks amongst captive waterfowl have been described in Israel [28] and North America [29,30,31,32], with high seropositivity in domestic geese (*Anser anser domesticus*) and Canada geese (*Branta canadensis*) in the outbreak in 1999 [33]. However, it is unclear whether domestic ducks (*Anas platyrhynchos domesticus*) and geese can produce viremia levels that are high enough to infect mosquitoes. Furthermore, it is also uncertain whether horizontal WNV transmissions (i.e., in-contact transmissions) can occur within commercial flocks of geese, as observed in experimental settings [34,35]. The relevance of WNV to poultry was demonstrated in infection studies, where, for example, domestic chickens (*Gallus gallus domesticus*) were susceptible to WNV, but did not develop viremia sufficient to infect mosquitoes [36]. 

In North America, captive or free-ranging bird species are frequently used as sentinels to detect and monitor arbovirus transmissions [37]. For example, chickens and pheasants (*Phasianus colchicus*) have been used for the surveillance of multiple avian arboviral encephalities, such as WNV [38], Western equine encephalomyelitis, and St. Louis encephalitis [39]. Candidate sentinel animals for WNV must be susceptible to a WNV infection and rapidly seroconvert, most suitably without contributing to the local transmission cycle (i.e., low viremia levels and viral shedding) [37].

This study evaluated the susceptibility of different domestic avian species (chickens, ducks, and geese) to better assess the risk WNV poses to German poultry. An Italian WNV lineage 1 strain [40] was selected for the experiments as, until the summer of 2018, it had been postulated that WNV would most likely enter Germany from the south. Only now, with the current German WNV situation, was this speculation refuted as the first German isolate was probably introduced from the Czech Republic [41]. All three poultry species were inoculated via needle injection and examined with respect to the degree and duration of viremia, oropharyngeal and cloacal viral shedding, tissue tropism, and seroconversion. However, in order to accurately reproduce the natural transmission cycle, geese were also exposed to infected mosquitoes, being the most susceptible to WNV after needle injection. It is important to estimate the extent of mosquito saliva-induced modulation of WNV pathogenesis in that specific species in order to assess the role a poultry species plays in the enzootic transmission cycle (as reservoir or amplifying host). This study assists in clarifying whether free-ranging commercial poultry can constitute a virus reservoir and be a potential hazard for public health. Finally, the suitability of chickens, ducks, and geese as candidate sentinels was also investigated. 

## 2. Materials and Methods

### 2.1. Ethical Approval

All of the infection experiments were performed according to biosafety level 3 regulations and in view of animal welfare, following national and European legislation, in particular, directive 2010/63/EU. The experiments were approved by the State Office of Agriculture, Food Safety, and Fishery of the federal state of Mecklenburg-Western Pomerania, Germany (Reference number 7221.3-1.1-018/18, approved 10 April 2018).

### 2.2. Domestic Poultry

Except for the chickens, which derived from specific pathogen-free (SPF) hatching eggs (VALO BioMedia, Osterholz-Scharmbeck, Germany), all of the animals were purchased as 1- or 2-day-old hatchlings from local poultry farms (Table 1). All birds were tested to be free from a WNV infection and, additionally, from salmonellosis (e.g., an infection with *Salmonella typhimurium*) prior to being subjected to the pathogenesis studies.

### 2.3. Virus

The WNV lineage 1 strain (TOS_09; GenBank accession no. HM991273/HM641225) was originally isolated during an outbreak in 2009 from the serum of a patient with neuroinvasive illness living in Italy [40]. After multiple passages in Vero E6 [40] and Vero ATCC cells [42], the virus was passaged once on *Aedes aegypti* C6/36 and once on Vero 76 cell monolayers. For this purpose, the cells were kept in modified minimum essential medium (MEM) that was supplemented with 2% fetal calf serum (FCS) (collection of Cell Lines in Veterinary Medicine, Friedrich-Loeffler-Institut (FLI), Germany). Two separate virus stocks, one for the animal infections and one for the mosquito injections, were harvested three days post-infection (dpi) and kept at −70 °C in 500 µL aliquots at final concentrations of 10^7.6^ and 10^8.3^ 50% tissue culture infective dose (TCID_50_) per mL, respectively. Virus was quantified by means of an endpoint dilution assay on Vero 76 cells, and the virus titer was calculated with the Spearman–Kaerber algorithm [43].

### 2.4. Mosquito Strain

Adult *Culex pipiens* biotype *pipiens* females (F0) were derived from egg rafts field-collected in the federal state of Brandenburg (in the areas of “Groß Kreutz”, “Schöneiche”, and “Rehfelde”), Germany. For their identification to species and biotype, hatching larvae (1–3 per egg raft) were analyzed by a real-time polymerase chain reaction (PCR) [44]. After pupation, the pupae were transferred into mosquito breeding cages (BugDorm; MegaView Science Co., Ltd., Taichung, Taiwan), and emerging adult mosquitoes were offered a 5–6% sugar solution ad libitum. They were maintained at 24 ± 1 °C with a relative humidity of 60–70% and a 16 h light: 8 h dark photocycle. A minimum of two non-engorged females per population were examined via a WNV-specific reverse transcription quantitative real-time PCR (RT-qPCR) in order to confirm that the field-collected mosquito populations were free from WNV prior to the experiments [45].

### 2.5. Subcutaneous Injections

After seven days of acclimatization, eight juvenile (approximately 3.5-week-old) chickens, ducks, and geese were infected subcutaneously (s.c.) with the Italian WNV lineage 1 strain. Each bird was inoculated, on average, with 10^6.0^ TCID_50_ of WNV diluted in 500 µL of MEM (250 µL in each knee fold). Another study demonstrated that the median WNV dose inoculated by *Cx. pipiens* into a host during probing and feeding was 10^6.1^ plaque forming units (PFU) [46]. Therefore, we hypothesized that German mosquitoes would inoculate a similar amount of WNV and chose a viral dose of 10^6.0^ TCID_50_ for a better comparison between birds infected via needle and those infected via mosquito bite. The birds were identified via numbered color-coded bands (chickens, ducks, and geese no. 1–8 for infected) and kept per species together in a pen. In addition, four animals per species were kept as a control group and housed in a separate pen (chickens, ducks, and geese no. 9–12 for controls). All of the birds were provided with water and food ad libitum. Waterfowl had access either to a small swimming pool or a small water basin for plumage care.

### 2.6. Mosquito Bite Challenge

Adult *Culex pipiens* biotype *pipiens* females (F0), up to 14 days old, were anesthetized with 100% carbon dioxide (CO_2_) in groups of ten and intrathoracically injected with the Italian WNV lineage 1 strain to guarantee that each individual mosquito would be WNV-positive. Procedures were performed under BSL 3 conditions inside a glove box and under microscopic control with a nanoliter injector (Nanoject II; Drummond Scientific Company, Broomall, PA, USA) and finely pulled 5 µL capillaries (PUL-1000 Micropipette Puller; World Precision Instruments, Sarasota, FL, USA). The mosquitoes were injected into the thorax with 41.4 nL of approximately 500 TCID_50_ and subsequently transferred into *Drosophila* tubes (Carl Roth, Karlsruhe, Germany) [47]. They were kept in an incubator (MLR-352H-PE; Panasonic Corporation, Osaka, Japan) at 25 ± 1 °C with 80–85% relative humidity and a 16 h light: 8 h dark photocycle. During this time, they had access to a 5–6% sugar solution. Nine days post injection and 24 h prior to the infection of the birds the surviving mosquitoes were sorted into groups of 8–13 specimens per modified 60 mL container (screw cap on one end and netting on the other; Thermo Scientific^TM^ Samco^TM^, Waltham, MA, USA) with no access to water or sugar solution. 

Infection of 3.5-week-old geese was performed late in the afternoon under dimmed light conditions. Each goose was manually fixated, and a small breast area was covered with a thin layer of goose blood mixed with adenosine triphosphate (ATP) as a phagostimulant and allowed to dry. Subsequently, each goose was held above a mosquito container for 30 min. During the feeding process, mosquitoes were regularly monitored and the blood engorgement status was noted afterwards.

### 2.7. Vector Competence of Mosquitoes Used in the Pathogenicity Study

One day after challenging the geese with WNV-infected mosquitoes, salivation assays were performed with the surviving mosquitoes. The mosquitoes were immobilized by removing their legs and wings under 100% CO_2_-anesthesia in preparation for the forced salivation assay, performed according to Heitmann and colleagues [48]. After mosquitoes had expectorated saliva, their heads were dissected from their bodies (thorax and abdomen). Mosquito heads, bodies, and legs plus wings were placed into separate 2 mL screw cap tubes with two 3-mm steel beads and 560 µL AVL viral lysis buffer and carrier RNA (Qiagen, Hilden, Germany) and stored at -70 °C until RNA extraction. Saliva samples were inoculated directly onto Vero 76 cell monolayers to detect viable and replicable virus. If a cytopathogenic effect was observed seven days after inoculation, cell culture supernatant was removed for the extraction of nucleic acid with the QIAamp Viral RNA Mini Kit (Qiagen). All other mosquito samples (i.e., mosquito heads, bodies, and legs plus wings) were first homogenized (two minutes at 30 Hz; TissueLyser II; Qiagen) and nucleic acid was then extracted with the BioSprint 96 (Qiagen) using the NucleoMag VET Kit (Macherey-Nagel, Düren, Germany). For a detailed description of the procedure see Holicki et al., 2020 [49]. All samples were screened by a WNV-specific RT-qPCR assay [45] with an additional calibration curve based on synthetic RNA for the quantification of viral copies. For this purpose, an AgPath-ID One-Step RT-PCR Kit (ThermoFischer, Darmstadt, Germany) and the CFX96^TM^ Real-Time PCR Detection System (Bio-Rad Laboratories, Feldkirchen, Germany) were used. The infection and dissemination efficiencies describe the number of mosquitoes with virus-positive bodies (thorax and abdomen) and legs plus wings, respectively, among those tested, while transmission efficiency refers to the presence of virus-positive heads and/or salivary secretions among those tested.

### 2.8. Sample Collection

The birds were monitored daily by a veterinarian according to a clinical score sheet: from 0 (no clinical signs) to 3 (severe clinical signs). Observations focused on behavior, body posture, plumage, eyes/conjunctiva, feed/water uptake, quality of feces, nutritional status, and neurological or respiratory signs (Supplementary data Table S1). To check for a steady gain in weight, the birds were weighed two days prior to infection and from 1 to 8, and 10, 14, 19, and 20/21 dpi. Blood and cloacal and oropharyngeal swab samples were also taken from all birds two days prior to infection (−2 dpi) in order to verify that the birds were WNV-negative before the infection. During the predicted viremic phase, from 1 to 6 dpi, blood samples were taken (from the ulnar or media metatarsal vein) daily from two alternating groups. The aim was to reduce the stress level for each individual bird and to enable sufficient convalescence between blood collection dates. To confirm seroconversion in the birds, further blood samples were collected from all birds 10 and 14 dpi, with final blood taken 20/21 dpi from the jugular vein. All of the blood samples were centrifuged at 2500 or 3500 rpm for 10–15 min and frozen away at −70 °C as serum and blood cruor or plasma.

Cloacal and oropharyngeal swabs were collected from 1 to 8, and 10, 14, and 20/21 dpi to assess viral shedding. The swab ends were placed into sterile polypropylene tubes (Greiner Bio-One, Frickenhausen, Germany) containing 2 mL of MEM supplemented with antimicrobials (gentamicin, amphotericin B, lincomycin, and enrofloxacin). Swabs were shaken at room temperature for 30 min at 50 rpm (Duomax 1030; Heidolph Instruments, Schwabach, Germany) and the supernatant was transferred into 2 mL tubes for storage at −70 °C until RNA extraction.

Necropsy was performed on all birds, including those that succumbed (one duck 2 dpi) or had to be euthanized (one goose 19 dpi and one goose 20 dpi) prior to the termination of the experiments 20/21 dpi. Tissue samples (including inter alia: brain, liver, spleen, heart, and bursa cloacalis) were collected while using multiple sterile scalps and forceps per bird to avoid cross-contamination between organs. They were stored in 7 mL screw cap tubes (Tube 7 mL 47x20PC+Cap; Sarstedt, Nümbrecht, Germany) at −70 °C for virological examination or in 4% neutral buffered formalin for histopathological investigation. 

### 2.9. Serology

The serum samples (from −2, 10, 14, and 20/21 dpi) were heat-inactivated (30 min at 56 °C) prior to analyzing them with a virus neutralization test (VNT) on Vero 76 cells. Consecutive dilutions (1:10, 1:20, 1:40, etc.) of the samples in MEM were challenged with 100 TCID_50_ of the homologous WNV strain from Italy and were conducted, as described by Seidowski et al., 2010 [50]. Neutralizing titers were determined with the Behrens–Kaerber method [51] after formalin fixation and crystal violet staining of the cell monolayers. For confirmation, the samples were also screened in the ID Screen^®^ WN competition enzyme-linked immunosorbent assay (ELISA) (IDVet, Grabels, France) according to the manufacturer’s instructions.

### 2.10. Reverse Transcription Quantitative Real-Time PCR

Pin-sized blood cruor and tissue samples were homogenized (two minutes at 30 Hz; TissueLyser II; Qiagen) in 600 µL RNeasy Lysis Buffer (RLT) (Qiagen) plus 6 µL ß-mercaptoethanol or 500 µL of MEM plus antibiotics (penicillin/streptomycin), respectively, with the addition of one 5-mm steel bead. Viral RNA was extracted from the blood cruor supernatant using the RNeasy Mini Kit (Qiagen), according to the manufacturer’s instructions. For the organ and swab samples, 100 µL of supernatant were used to extract nucleic acid with the BioSprint 96 (Qiagen) using the NucleoMag VET Kit (Macherey-Nagel). All of the RNA extracts were examined with the specific WNV RT-qPCR assay targeting the 5′ untranslated region (5′ UTR) for the simultaneous detection of lineage 1 and 2 strains [45]. For the quantification of viral RNA copies in each sample, a calibration curve of synthetic WNV-RNA was run in parallel using 10-fold serial dilutions [45]. Therefore, viral load is always given as viral RNA copies/µL of the total RNA volume, tested via RT-qPCR. An additional universal internal control was also used [52].

### 2.11. Histopathology and Immunohistochemistry (IHC)

At necropsy, tissue samples were fixed in neutral buffered formalin (4%). After being embedded in paraffin, 3 µm sections of the tissue blocks were mounted on Superfrost plus slides (Menzel-Gläser, Braunschweig, Germany), deparaffinized, and rehydrated. For histopathological examination, the sections were stained with hematoxylin/eosin (HE). In addition, IHC was performed with representative samples that were WNV-positive in the RT-qPCR (i.e., brain and spleen). Pretreatment included blocking of endogenous peroxidase with 3% H_2_O_2_/methanol, a proteinase K (Roche, Mannheim, Germany) digestion (15 min at 37 °C with 4 µg/mL) in order to retrieve the antigen, and a serum block directly before incubation with the antibodies. As a primary antibody, the in-house polyclonal antibody OM8 was used for the detection of WNV antigen at a dilution of 1:1,700 in goat serum. Negative control sections were only incubated with goat serum. For development, the EnVision^TM^ reagent (Dako Diagnostics, Hamburg, Germany) was applied, followed by diaminobenzidine-tetrahydrochloride staining and counterstaining with Mayer’s hematoxylin.

### 2.12. Data Analysis

Data were visualized with R version 3.6.0 (26 April 2019) [53] and LaTex and assessed biostatistically (wherever it was sensitive). 

## 3. Results

### 3.1. Clinical Signs during Infection

Except for the geese, none of the tested avian species displayed WNV-associated clinical signs. Food and water intake remained normal, and all of the birds steadily gained weight throughout the three weeks post infection. One duck (D 07) succumbed 2 dpi without showing clinical signs. Two of the eight geese that were infected via subcutaneous injection showed clinical signs in the third week after infection. These signs were nonspecific and included lethargy, reduced appetite/inappetence with body weight loss, impaired movement, skewed body posture, temporary muscle tremor, and ruffled and dirty plumage. After reaching the maximum allowed clinical score (Supplementary data Table S1), the two birds were euthanized (19 and 20 dpi). 

### 3.2. Infection Profile of Poultry Infected via Subcutaneous Injection (Viremia and Viral Shedding)

Viremia was detected in the blood of all three tested poultry species and it was quantified as the amount of WNV-specific RNA determined as copies per µL of total RNA (Supplementary data Table S2). The chickens had the lowest viremia levels, with viremia detectable only for one day (2 dpi). The viral load of the ducks and geese peaked 3 and 2 dpi, respectively, reaching up to 19.4 and 36.3 viral copies per µL of total RNA. In the ducks, WNV antigen was detectable for two consecutive days (2 and 3 dpi), while, in the geese, virus RNA was measured from 2 to 6 dpi. In contrast, one goose (G 08) developed no viremia after subcutaneous injection of WNV. The blood samples that were taken prior to WNV-infection and those of all control animals were WNV-negative. 

Viral shedding was observed in the chickens, ducks, and geese from 2 to 5 dpi. Similar to the low viral load in the blood, the chickens only had small quantities of WNV-specific RNA in their oropharyngeal swabs (cycle threshold (Ct) of 31.9–35.8 and 0.3–5.7 viral copies/µL of total RNA). The oropharyngeal shedding of the ducks was also fairly low (Ct 30.7–36.1 and 0.2–9.1 viral copies/µL of total RNA). Interestingly though, this was the only species with predominantly cloacal (Ct 29.6–36.1 and 0.2–19.8 viral copies/µL of total RNA) rather than oropharyngeal shedding. Geese shed the most virus and, similarly to the chickens, primarily shed oropharyngeally. The oropharyngeal and cloacal swab samples taken prior to WNV-infection and of all control animals were WNV-negative.

### 3.3. Infection Status of Mosquitoes after Intrathoracic Injection with WNV

Of 215 *Culex pipiens* biotype *pipiens* females intrathoracically injected with WNV 134 females (62.3%) survived until 9 dpi. After goose exposure (10 dpi), 99 mosquito females (46.0%), and in the in vitro transmission assay [49] (11 dpi) 92 females (42.8%), were still alive. These revealed infection and dissemination efficiencies of 100% as WNV-specific RNA was detected in all tested bodies (thorax and abdomen) and legs plus wings (Table 2). All head samples, but only 46 (50.0%) of the saliva samples, were tested virus-positive. The mean viral loads (WNV-specific RNA copies/µL of total RNA) were highest in the bodies (9.1 × 10^5^), followed by the heads (1.8 × 10^5^) and legs plus wings (2.9 × 10^4^). 

### 3.4. Infection Profile of Geese Infected via Mosquito Bite (Viremia & Viral Shedding)

**Figure 1:**
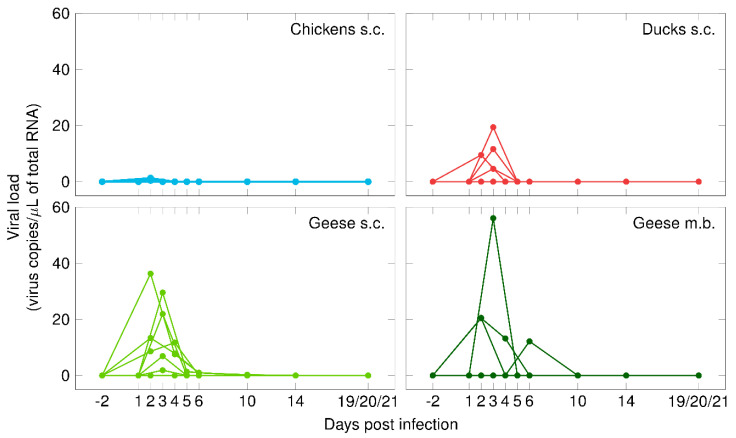
Viremia in chickens (in blue), ducks (in red), and geese (in green) after subcutaneous injection (s.c.) with WNV or the bite of WNV-infected mosquitoes (m.b.).

**Figure 2:**
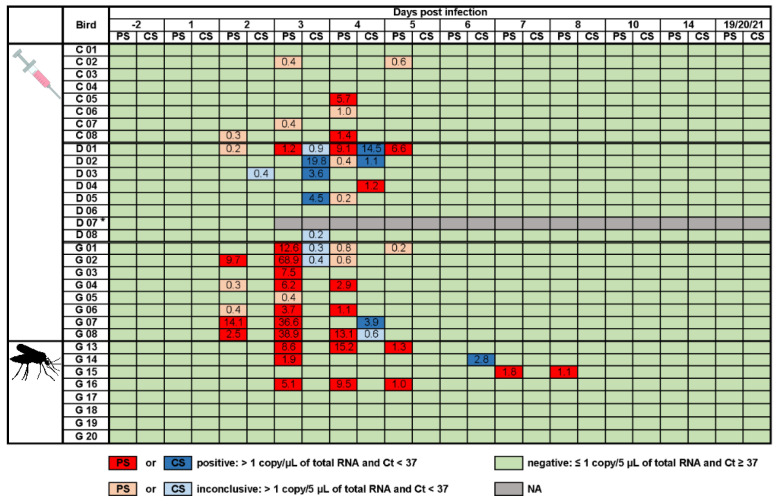
Viral shedding of chickens (C), ducks (D), and geese (G) after subcutaneous injection with WNV or the bite of WNV-infected mosquitoes. Numbers indicate the viral copies/µL of total RNA of positive or inconclusive oropharyngeal (PS in dark or light red) and cloacal (CS in dark or light blue) swabs. Asterisk indicates one duck (D 07) that succumbed early after infection, so that swab samples could only be taken one and two days post infection. NA stands for not applicable. Ct stands for cycle threshold.

Of the eight geese that were exposed to the WNV-positive mosquitoes, four became infected and seroconverted (G 13–G 16). Only one mosquito appeared to have imbibed blood (i.e., visual engorgement in the midgut), in this case from goose G 16. The absence of visible blood meal residues in the other mosquitoes indicates that the majority of the geese were presumably already infected during mosquito probing. Figure 1 shows the mean viral loads in the blood samples that were collected from all the birds. Of the geese that became infected via mosquito bite, the highest viral load (56.1 viral copies/µL of total RNA) was reached 3 dpi and was higher than the highest viral load (36.3 viral copies/µL of total RNA) reached by a subcutaneously infected goose 2 dpi (Supplementary data S2). In one goose (G 15), the first detection of WNV-specific RNA (6 dpi) was notably later than in the other geese. Additionally, the onset of viral shedding of goose G 15 was delayed (began 7 dpi) as compared to those infected via subcutaneous injection. Furthermore, the quantity of virus shed via the oropharynx was lower after mosquito bites (Ct 30.0–33.9 and 1.0–15.2 viral copies/µL of total RNA) than after needle injections (Ct 28.2–36.6 and 0.2–68.9 viral copies/µL of total RNA) (Figure 2). The remaining four geese from this infection trial were consistently tested WNV-negative in their blood and swab samples (oropharyngeal and cloacal). 

### 3.5. Serology

**Figure 3:**
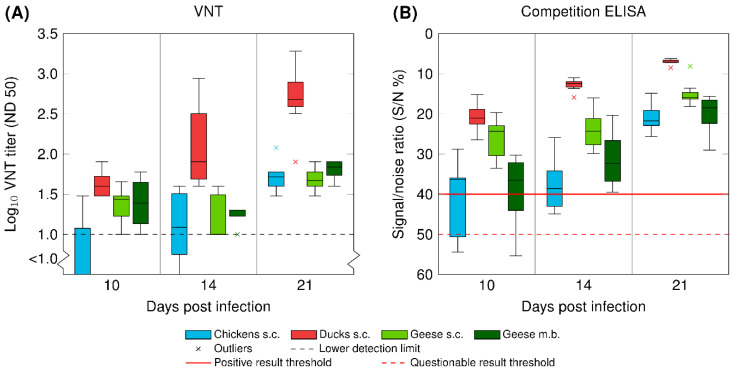
Seroconversion of chickens (in blue), ducks (in red), and geese (in green) after inoculation with WNV via subcutaneous injection (s.c.) or the bite of WNV-infected mosquitoes (m.b.; G 13 – G 16). (**A**) Antibody titers determined by a virus neutralization test (VNT) and (**B**) antibody titers determined by a commercial competition ELISA. Data are presented in a box-and-whisker plot, where the ends of the whiskers represent the minimum and maximum values. Outliers are represented by an x instead of the whisker-ends. The box includes 50% of the values of each group and the line in the middle of each group represents the median value.

Seroconversion was observed in all three poultry species after needle injection. Figure 3 and Supplementary data Table S3 depict the antibody levels 10, 14, and 19/20/21 dpi, measured via VNT and ELISA, with an overall increase in antibody titers over time. The fastest seroconversion was observed in the ducks with noticeably higher antibody levels 14 and 19/20/21 dpi. From the geese infected via mosquito bite, four seroconverted (G 13–G 16), while the others were deficient of WNV-specific antibodies throughout the experiment (G 17–G 20, data not included in Figure 3). Prior to the experiments, none of the birds had antibodies against WNV, and all of the control animals remained negative throughout the three weeks. 

### 3.6. Tissue Tropism of WNV

**Figure 4:**
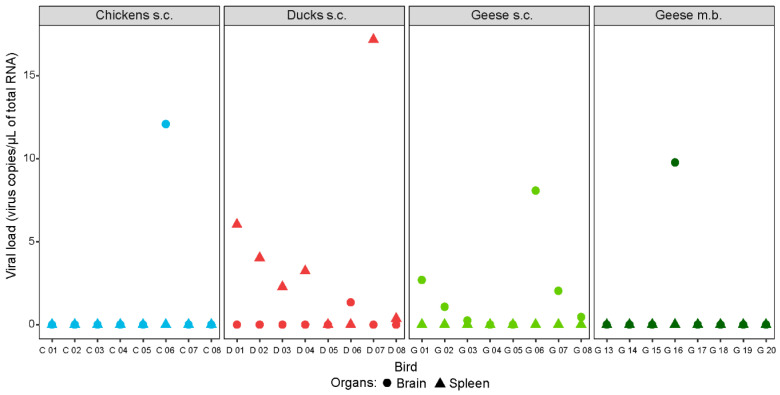
Selected organ samples of chickens (in blue), ducks (in red), and geese (in green) infected with WNV via subcutaneous injection (s.c.) or the bite of WNV-infected mosquitoes (m.b.). Brain samples depicted as filled circles and spleen samples as filled triangles. All birds were euthanized 19, 20, or 21 dpi, except for one duck (D 07) that succumbed 2 dpi.

Only a few isolated organ samples of the tested poultry species contained WNV-specific RNA 19/20/21 dpi (Figure 4 and Supplementary data Table S4). Tissue tropism varied between species, but not between inoculation methods (i.e., subcutaneous injection or mosquito bite). For example, after needle injection, virus RNA was detected solely in the brains of the chickens (1/8) and the brains (6/8 and 1/4) and bursa cloacalis (2/8 and 0/4) of the geese, but in various organs (preferred site: spleen, 6/8) of the ducks. The premature death of one of the ducks (D 07) 2 dpi, is potentially the reason for the detection of viral RNA also in the liver, heart, and skin of the injection site. Of the four geese infected via mosquito bite, only one organ sample (brain) was tested positive with 9.8 WNV-specific RNA copies/µL of total RNA. The organs of all control birds as well as of the four geese that had not been infected via mosquito bite were WNV-negative.

### 3.7. Gross Lesions, Histopathology, and Immunohistochemistry

Gross lesions were not observed in the chickens, neither in the negative controls nor in the infected animals. However, the histopathology revealed, in two of the infected birds (2/8), a multifocal mild to moderate acute to subacute non-suppurative encephalitis consisting of multifocal lymphohistiocytic perivascular cuffing with participation of a few plasma cells (2/2), glial nodules (2/2), scattered neuronal single cell necrosis (2/2), endothelial hypertrophy (1/2), and leucocytic degeneration of the migrating infiltrates (2/2). Lesions in the brain had a multifocal distribution, yet the brain stem was the most affected. One of these birds also displayed mild follicular hyperplasia in the spleen. Secondary findings were seen in all of the examined chickens, including control birds, and they are summarized in Supplementary data Table S5. 

Gross lesions were not seen in the control ducks. By contrast, most of the infected ducks (7/8) had a pale myocard, except for one duck (1/8) that died 2 dpi and had developed mild follicular hyperplasia of the spleen instead. In most of the animals (6/8), the histopathology revealed an oligofocal mild subacute non-suppurative encephalitis. Alterations were noted in all regions of the brain, with cerebrum (5/6) and cerebellum (5/6) being most affected and the brain stem (3/6) and mesencephalon (2/6) to a lesser degree. The most prominent histopathological findings in the brain included oligofocal lymphoplasmacellular perivascular cuffing (6/6) with leucocytic degeneration (4/6), scattered single neuronal cell necrosis (1/6), and glial nodules (6/6). Furthermore, several ducks (6/8) showed an oligofocal mild subacute interstitial myocarditis (4/6), while two ducks (2/6) exhibited an oligofocal mild necrotizing myocarditis (2/6). Only a few ducks (3/8) displayed alterations in the spleen: in two birds (2/3) mild follicular hyperplasia and in one (1/3, a duck that died 2 dpi) a distinct necrotizing splenitis. Secondary findings in the ducks, including negative controls, are summarized in Supplementary data Table S5. 

**Figure 5:**
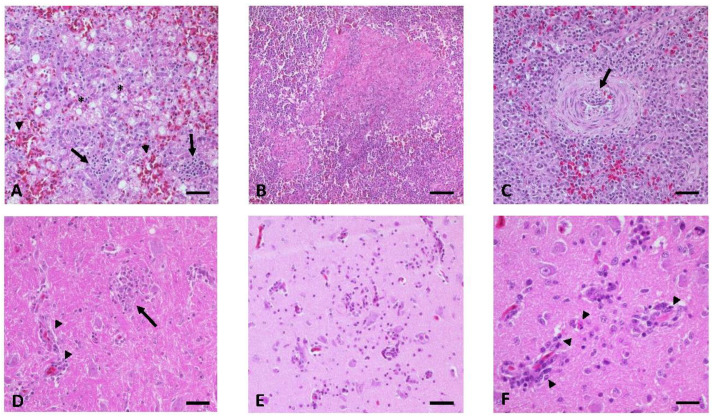
Histopathological findings in WNV-infected geese. (**A**) Liver of G 01, showing vacuolar degeneration (*), acute hemorrhages (arrowheads), and multifocal subacute non-suppurative hepatitis (arrows). (**B**,**C**) Variability of pathomorphological lesions in the spleen with (**B**) focal acute necrotizing splenitis of G 03 and (**C**) focal mild subacute non-suppurative arteritis with distinct intima hyperplasia (arrow) in the vessel wall of G 13. (D-F) Multifocal acute to subacute non-suppurative encephalitis (**D**) in the brain stem of G 02 with glia nodules (arrow) and mild lymphohistiocytic perivascular cuffing (arrowheads). (**E**) Cerebrum of G 06 with necrotic foci consisting of mild lymphoplasmacellular perivascular cuffing, migrating mononuclear cells, small glia nodules, and scattered single cell necrosis (neurons, glia cells, and leucocytes). (**F**) Cerebrum of G 06 with leucocytic degeneration (arrowheads) within the mononuclear perivascular cuffing (lymphocytes, histiocytes, and plasma cells). Bars: A–E 50 µm, F 20 µm.

As in the other domestic species, the control geese showed no gross lesions. However, the geese infected s.c. with WNV displayed several alterations, including a pale and mottled myocard (3/8), with additional multifocal acute hemorrhages in one goose (1/3). Furthermore, mild subcapsular acute hemorrhages (3/8) and focal necrotic foci (1/8) were seen in the liver. One goose, which had been euthanized before the termination of the experiment (19 dpi) after displaying clinical signs, had multifocal erosive to ulcerative dermatitis at both elbow joints and acute multifocal to coalescing subcutaneous hemorrhages, in particular, around the injection site. Histopathological lesions were present in the brains of all infected geese (8/8), with an acute to subacute non-suppurative encephalitis (Figure 5D–F) of various degrees, ranging from oligofocal weak (3/8) and mild (3/8) to multifocal and moderate (2/8). Cerebrum (7/8) and mesencephalon (7/8) were primarily affected, whereas brain stem (6/8) and cerebellum (4/8) were involved to a lesser degree. Brain lesions consisted of oligo- to multifocal perivascular lymphohistiocytic or lymphoplasmacellular cuffing, partly with distinct leucocytic degeneration (7/8), scattered necrosis of single neurons/glial cells (8/8), glial nodules (5/8), which were in one animal (1/5) associated with distinct neuronal necrosis, endothelial hypertrophy (3/8), and the degeneration of individual Purkinje cells (3/8). Further findings included a necrotizing splenitis (2/8) (Figure 5B) and mild to moderate follicular hyperplasia of the spleen (5/8). Livers displayed variable patterns of lesions, with oligofocal subcapsular acute hemorrhages (5/8, but also observed in the control geese), interpreted as early signs of a fatty liver disease. In three infected animals, these lesions were not only associated with hepatocellular vacuolar degeneration (3/5), but also with an additional non-suppurative hepatitis (1/3) (Figure 5A) or focal necrosis (1/3). One goose even developed an oligofocal mild intralobular necrotizing hepatitis (1/8). Supplementary data Table S6 summarizes additional secondary findings and findings in the negative control birds.

Similar findings were seen in the gross and the histopathological examination of the four geese infected via mosquito bite (4/8). All of the animals (4/4) revealed signs of an oligo- to multifocal mild to moderate, acute (1/4) to subacute (3/4), non-suppurative encephalitis with variable distribution of the foci throughout the brain. For example, in one goose all brain regions were affected (1/4), while in another (1/4) only the cerebrum was impacted. Inflammatory lesions of the brain were similar to those that were described for the subcutaneously infected group, except that one goose infected via mosquito bite (1/4) displayed focal necrotic foci. During gross examination, two of the geese (2/4) had a pale myocard that was associated with an oligofocal mild interstitial non-suppurative myocarditis. Furthermore, several birds (3/4) appeared to have undergone mild (1/4) to moderate (2/4) follicular hyperplasia of the spleen. Two of these birds had additional alterations in the spleen: an oligofocal fibrinonecrotizing vasculitis (1/4) and a multifocal subacute non-suppurative arteritis with distinct intima hyperplasia and necrosis of individual cells in the tunica media (1/4) (Figure 5C). A focal mild acute necrotizing enteritis with fibrinonecrotizing vasculitis was also prominent in one of the geese (1/4). The non-infected geese of this group showed no comparable alterations. Supplementary data Table S6 shows a summary of all results. 

The immunohistochemical examination of the spleen and brain of all three species (independent of the infection method) failed to detect viral (WNV) antigen.

### 3.8. Summary of Results

All three poultry species were susceptible to the Italian WNV lineage 1 strain and developed similar systemic infections with seroconversion and various degrees of viremia and viral shedding. Several birds from each species developed pathomorphological alterations that were typical for a viral infection. These were most frequent in the geese, followed by the ducks, and rare in the chickens. Furthermore, two geese developed WNV-associated clinical signs, in this case after subcutaneous injection. Independent of the infection method, all three species reached peak viremia 2–3 dpi, with the geese developing the highest viral loads. Predominantly oropharyngeal viral shedding was observed in the chickens and geese, while, in the ducks, shedding took place mainly cloacally. Of the eight geese exposed to the WNV-infected mosquitoes, four became infected. Geese infected via mosquito bite had the highest viremia levels and slightly more widespread and variable pathomorphological lesions in contrast to those infected via subcutaneous injection. Yet, the fairly low viral load in the blood of all experimentally infected birds would probably not suffice to pass on the virus to feeding mosquitoes. All three poultry species seroconverted, with ducks developing the highest antibody titers. The tissue tropism varied between the species, with WNV mainly infecting the brains of chickens and geese and the spleens of ducks. Unfortunately, antigen detection by IHC was unsuccessful. 

## 4. Discussion

In the recent years, WNV led to annually recurring enzootic and epizootic outbreaks in southern, southeastern, and lately also central European countries [54]. Therefore, it deemed only a matter of time until WNV would emerge also in Germany. Eventually, an incursion of WNV was confirmed in 2018 with virus isolation from two horses and 12 captive or wild birds, such as great grey owls (*Strix nebulosa*), northern goshawks (*Accipiter gentilis*), and blackbirds (*Turdus merula*) [41,55]. In the year 2019, an already much larger number of birds (*n* = 76), horses (*n* = 36), and even mosquito pools (*Cx. pipiens*, *n* = 7), as well as humans (*n* = 5) were tested positive for WNV [55,56]. These cases were locally confined to the east of Germany, where warm weather conditions prevailed [41,55]. 

At that time, it was difficult to assess which amplifying hosts would assist in perpetuating and spreading WNV and which species are best suited as sentinels for passive and active virus monitoring. Even though WNV has been reported in domestic and free-ranging birds all over the globe, their susceptibility is known to vary considerably, depending on virus strains and old world versus new world avian species [27]. Experimental infection studies using local virus strains and indigenous bird species are, therefore, needed to estimate the role certain avian species play in the local enzootic transmission cycles. Published experimental research has focused predominantly on the susceptibility of American avian species and American virus strains (e.g., NY99) [27]. In Europe, isolated infection experiments have been performed with local domestic bird species (e.g., red-legged partridge (*Alectoris rufa*) in Spain [57,58], rock pigeons (*Columba livia*) in Italy [59], or SPF chickens in France [60,61]) while using circulating WNV isolates. 

By comparison, this present study aimed at uncovering the role domestic avian species might play in Germany for the transmission cycle of a relevant European WNV lineage. The birds were infected with an Italian WNV isolate at a time when WNV had not yet been isolated in Germany and indigenous mosquito species (*Cx. torrentium* and *Cx. pipiens*) had only been proven vector-competent for this exact European isolate [42]. Our study, therefore, focused on this specific Italian WNV lineage 1 strain and on poultry bred in Germany: chickens, ducks, and geese. Poultry farming is an important part of Germany’s economy, with the production of 1.8 million tons of meat, primarily broilers, in 2018 and a per capita consumption of 22.2 kg [62]. 

In our study, domestic chickens, ducks, and geese were susceptible to infection with the Italian WNV lineage 1 strain. Even though most of the birds did not develop clinical signs, all had viremia levels that peaked 2 or 3 dpi, shedded virus from 2 to 8 dpi, and rapidly seroconverted. The geese appeared to be most susceptible, with the highest viremia levels and most frequent pathomorphological alterations. Similar results were achieved in the geese that were infected via mosquito bite. To some extent, these results coincide with several epiornitics described in geese as, for example, in the USA [31], Canada [29], Israel [28,63], and Hungary [64]. In addition, infection experiments described that the neurovirulent, phylogenetically closely related WNV strains from the outbreaks in New York (1999) [18] and in Israel (1998) [65], were associated with severe neurological signs, mortality, and direct transmission in geese [34,35,63,66,67]. The results in this study showed that the Italian WNV isolate, unlike strains from New York or Israel, caused WNV infections without mortality in German geese. Even though these geese sustained distinct histopathological lesions related to a virus infection, there is insufficient evidence supporting a similar pathogenicity of European WNV strains. This could be due to a reduced pathogenicity of the used European virus strain and/or a limited virus susceptibility of German avian species. Similarly, infection experiments with free-ranging European birds, such as European jackdaws (*Corvus monedula*) [68], carrion crows (*Corvus corone*) [69], and house sparrows [70], indicated reduced virulence of Italian WNV isolates in comparison to the New York, 1999 strain. Nonetheless, American crows (*Corvus brachyrhynchos*) showed 100% mortality rates and high viremia levels after WNV infection with Italian strains [71], suggesting a possible greater importance of host factors (genetic composition of certain breeds, immune response, and physiological mechanisms) than strain-dependent susceptibilities [69].

Understanding the varying immune responses to WNV among the tested avian species can aid in unraveling why the ducks developed the highest antibody levels, while the geese were most susceptible. Research in the past has focused on the immunocompetence of mammalian, primarily murine species (summarized by Bai et al., 2019 [72]). Although the course of infection in birds is also influenced by innate as well as adaptive immune responses, there are definite differences in functional mechanisms, as well as in genes, molecules, cells, and organs involved [73]. The innate immune response is the first line of defense and plays an important role in WNV disease development. Firstly, pathogen-associated molecular patterns (PAMPs) are recognized by pathogen-recognition receptors (PRRs), including Toll-like receptors (TLRs), retinoic-acid-inducible gene I (RIG-I)-like receptors (RLRs), and NOD-like receptors (NLRs). These receptors mediate antiviral immunity by triggering the production of type I interferon (IFN) and other cytokines [72]. Ducks, for example, can steadily replicate low pathogenic avian influenza (AI) viruses in their intestinal tissues and constitute as natural virus reservoirs. However, if infected with highly pathogenic AI, they can induce an immediate and robust type I interferon response (via the RIG-I pathway) [74]. Monocytes and macrophages belong to the cellular defense mechanisms and they are considered to be the primary targets of WNV and therefore, contribute to the spread of the virus throughout the tissues [17]. They can secrete high levels of type I IFN and of pro-inflammatory cytokines or they can act as specialized phagocytes, presenting antigen on their cellular surface [72]. Their relevance was proven for multiple corvid species by in vivo and ex vivo infection studies [75,76]. Unfortunately, it was not possible to compare the WNV target cells between the poultry species due to the lack of antigen detection in the IHC in this study. Studies have also focused on the adaptive immune response such as B- and T-cell activation and antibody production [17]. For example, the recognition of WNV non-structural protein 1 peptide epitopes varied between the antisera of immunized chickens, ducks, and geese [77]. Ducks in this study produced the strongest immune responses (i.e., highest antibody levels), with the spleen possibly playing a key role. As stated by Smith et al., 2004 [78], the spleen has an essential role in the avian immune system, as it is an antigen presentation and maturation site for lymphocytes, which are responsible for the cellular and humeral immune responses. The spleen was not only the dominant site for the detection of WNV-specific RNA in ducks in this study, but, unlike in the geese, the spleens showed no distinct histopathological lesions in all but one duck. This one duck rapidly developed a splenitis after infection and succumbed 2 dpi. This exception might have been due to existing individual differences influenced by still unknown factors like hormonal status, stress, or the presence of concurrent diseases [17]. However, as this was the first infection study performed with German WNV isolates and avian species, focus was primarily placed on the pathogenesis of the virus strain rather than on immune responses. Future studies should in-depth analyze the immune response of ducks and geese to WNV, as, to our knowledge, no immunological research in the context of WNV has been performed with these two species in the past. Useful techniques and approaches could comprise the sequencing of immune related genes in tissue cells [79], the flow cytometric assessment of lymphocyte subsets in the peripheral blood [80] and spleen, and the immunostaining of WNV antigen in the brain at early time points post infection to better understand neuroinvasion and neurodegeneration [72]. In particular, investigations focusing on the pathophysiology of the blood-brain barrier would help to uncover WNV neuropathogenesis. Even though the presence of perivascular inflammatory infiltrates in all three bird species in this study was indicative for the transportation of WNV to the brain via the blood stream, an additional detection of WNV antigen via IHC in endothelial cells would have assisted in undermining this theory. The transport of WNV through infected immune cells also remains plausible in avian species [17]. 

Although wild hatch-year birds are believed to support enzootic WNV amplification in Europe (i.e., transmit the virus to naïve mosquitoes during feeding) [81], it is unlikely that this applies to juvenile chickens, ducks, and geese. Their viremia levels did not reach the 10^6.0^ PFU/mL supposedly required to infect susceptible mosquitoes [5]. However, also in Europe, direct virus transmission between geese either due to viral shedding or cannibalism and feather picking cannot be ruled out. Direct transmission was not only confirmed for various wild bird species (blue jays, black-billed magpies, American crows, and ring-billed gulls) [82], but also for geese after the infection with the Israel 98 strain [35] and chickens infected with New York strains from 1999 [36] and 2000 [83]. Interestingly in this study, one goose (G 15) exposed to infectious mosquitoes showed delayed viremia and viral shedding, with WNV-specific RNA in its blood 6 dpi and oropharyngeal samples 7 and 8 dpi. Even though feather picking was observed, these findings should be interpreted with caution, as the delay in viremia and viral shedding is more likely due to variations in the infection process after a mosquito bite, rather than indicating a direct transmission. 

Only a few of the tissue samples from the birds euthanized 19/20/21 dpi were positive for WNV-specific RNA, with a noticeable discrepancy in the tissue tropism between the tested species. RNA was primarily isolated from the brains of chickens and geese in contrast to the spleens of ducks. The high prevalence in the spleens of ducks coincides with their greater immune response and it has already been described for Aigamo ducks [84]. It is presumed that the immune response of all tested poultry species rapidly and effectively cleared WNV from most of their organs (except brain and spleen), with subsequent development of histopathological lesions [85]. This also explains why more WNV-specific RNA was found in multiple organ samples (liver, spleen, and heart, but not brain) of a duck (D 07) that already succumbed 2 dpi. Histopathologically, this duck also displayed typical signs for an early viral (WNV) infection [17], with the manifestation of a massive splenic necrosis, but no encephalitis. However, even in this case, viral antigen was not detectable via IHC. This may have been due to the very early time point of necropsy (i.e., not yet existing brain lesions) in combination with the massive necrosis of the spleen, possibly having destroyed the necessary epitopes for the IHC. 

Distinct species differences regarding the histopathological lesions were also visible yet they did not necessarily correspond with the isolation of viral RNA from the organ samples.The heart was the most severely and frequently affected organ in ducks, in contrast to the brain and spleen of geese. Interestingly, necrotizing alterations, as commonly seen in other studies [17,86,87,88,89], were rare in both species. As has already been described for other virus strains [17], the chickens showed reduced susceptibility to WNV, with only two animals revealing mild lesions that were confined to the brain. On the other hand, in this species, a unique necrotizing component was observed. A common pattern in all species, also associated with other flavivirus infections, such as with TBEV [90], was the detection of a mild leucocytic degeneration, in particular of the migrating inflammatory cells within the perivascular cuffing. 

Unfortunately, the IHC could not detect viral antigen in any of the examined birds. This comes by no surprise, as the number of WNV-specific RNA copies were low in most cases and the time point of death/euthanasia was either very early or very late after onset of virus infection. Previous studies in falcons [91] already proved that there is only a finite period of time for the immunohistochemical detection of WNV-antigen in tissue samples. Nonetheless, as the observed pathomorphological lesions are indicative of a viral infection, the tissues involved are typical for a WNV infection (for review see Byas and Ebel 2020 [86], or results from experimental studies with chickens [89], ducks [84,92], and geese [34,35,66]), and none of the control animals showed comparable alterations, there is no doubt in the direct association of lesions with the experimental WNV infection. However, it could not be clarified what role virus infection played in the establishment of the diverse liver lesions. An indirect relation to stress and anorexia is conceivable, as exemplified by G 01 and G 03. These two geese are of special interest, as they were both euthanized towards the end of the experiment after having manifested a clinical disease a few days before. Histopathology revealed that both birds displayed clear signs of an acute viral infection with distinct necrosis in the spleen, a common pattern in certain avian species early on during the incubation period (for review see Gamino and Höfle 2013 [17]). Furthermore, unlike any of the other geese, both displayed necrotic liver lesions. A possible secondary infection with WNV is unlikely, as the blood samples displayed no evidence of another viremic episode at a later time. However, an explanation could be virus persistence, as has been described for numerous vertebrate hosts [93,94,95,96]. Stress or other yet to be determined factors could have possibly reactivated virus replication. The periodic recrudescence of persistent WNV infections in avian hosts, in addition to the overwintering of WNV in hibernating mosquitoes, could be a plausible explanation for the viral overwintering and reestablishment of vector transmission in the spring and summer months [97]. Further studies concerning the exact pathogenesis of WNV infections and the humeral and cell-mediated immunity in poultry are necessary and could not only further clarify some of the results seen here, but also improve and accelerate dead-bird surveillance programs. 

So far, this is the first study to show that German field-collected *Cx. pipiens* biotype *pipiens* populations, intrathoracically injected with WNV, can transmit the virus ten days after injection to immunologically mature geese (approximately 3.5 weeks old). The same mosquito populations from “Groß Kreutz”, “Schöneiche”, and “Rehfelde” have already proven to be capable of transmitting the first isolated German WNV strain (WNV lineage 2; GenBank accession no. MH924836) after feeding on virus-spiked blood under laboratory settings [47]. In combination with other studies [46], there is enough supportive evidence for the role of *Cx. pipiens* biotype *pipiens* in the WNV transmission cycle in Germany. 

The viremia levels of geese infected with WNV via the bite of infected *Cx. pipiens* biotype *pipiens* were slightly higher than those of the geese subcutaneously inoculated with 10^6.0^ TCID_50_/500 µL of WNV. The magnitude of viral shedding was not higher after infection by mosquito bite, only the onset of oropharyngeal shedding was, in part, delayed. However, the observed pathological lesions seemed to be more widespread and diverse with, for example, arteritis in one case and enteritis in another, as compared to the subcutaneously infected group. Other studies have also described an enhanced infection in mice [98,99] and chickens [83], with higher viremia and greater viral shedding, after infection via mosquito bite when compared to subcutaneous injection. It is suggested that saliva introduced at the site of infection can modulate the immune response of keratinocytes and local dendritic cells. During probing and feeding a mosquito ejects saliva into the host, which contains pharmacologically active components, known to modify, for example, cytokine levels and to lead to a dysregulation or suppression of the innate immunity [100]. Saliva can also increase the infiltration of inflammatory cells, such as leukocytes, thereby altering the composition of host cells and possibly supporting viral replication [101]. However, as in the majority of such experiments, it is difficult to determine whether observed differences between application types should be attributed to saliva components or methodological approaches. For example, methodological variances exist in the application site (intradermal vs. subcutaneous), the viral dose (exact amount of virus secreted by one or more mosquitoes is unknown, as is the potentiation effect of multiple mosquito bites [83,99]), and the viral source (invertebrate vs. vertebrate cells) [27,99]. It is probable that three of the four geese were infected merely by mosquito probing and that low viral quantities sufficed to infect the geese, as only one single mosquito appeared to be partially blood-fed after exposure to the geese. The remaining four geese that did not show any signs of infection were most likely not exposed to infectious saliva at all or only to an insufficient amount.

The three tested domestic avian species should be considered to be candidate sentinels for monitoring WNV circulation in Europe. After infection, they all rapidly seroconverted without producing blood viral loads high enough to infect mosquitoes. If placed directly into regions with known or suspected WNV enzootic cycles, they are likely to reliably indicate natural virus circulation and an imminent epiornitic, epizootic, or even epidemic outbreak [37]. Furthermore, sentinels should produce neutralizing antibodies soon after infection. Previous studies have confirmed that the rise of antibodies in chickens occurs between 5 and 10 dpi [36] and they are continually present in chickens, turkeys, ducks, and geese 14 dpi [36,66,89,92,102], as was the case in this study. Chickens have been used as sentinels in South Africa [103,104], Australia [105,106], Romania [107], Greece [108], and in multiple states in the USA, for example, in New York, in 1999, with an overall seroconversion of 63% [33,109]. However, the use of chickens in the USA was not as successful as presumed as seroconversion was often detected only after the onset of human infections [37]. The results of this study propose the use of free-ranging geese or ducks, rather than chickens. Especially, ducks could be well-suited as they produce high antibody titers and are a preferred blood-source for *Culex* mosquito populations, such as for *Cx. pipiens* biotype *pipiens* mosquitoes [110]. The implementation of the active monitoring of waterfowl in Germany is also feasible due to the annual free-range rearing of these species throughout the summer and their slaughter in late autumn enabling the easy collection of blood samples. This facilitates the establishment of monitoring networks, especially in high-risk WNV areas and their neighbouring regions. Well-developed monitoring strategies are essential for observing the spread of WNV and collecting epidemiological data for preventive healthcare. 

## 5. Conclusions

In conclusion, juvenile domestic avian species (chickens, ducks, and geese) bred in Germany are susceptible to a European WNV lineage 1 strain and they can be used as sentinel species in free-range production to assess ongoing WNV activities in the environment and, thus, can inform public health authorities in order to initiate precautionary actions and interventions. German poultry species were not confirmed to act as natural virus reservoirs. In this study, there are possible indications for an enhanced pathogenicity of WNV to its host in combination with salivary components of German *Cx. pipiens* biotype *pipiens* mosquitoes. Future studies are necessary with German free-range poultry and the currently circulating WNV lineage 2 strain to better evaluate the pathogenesis of the disease and current risk for human and animal health.

## Figures and Tables

**Table 1:** Domestic avian species infected with West Nile virus (WNV) lineage 1 and their number coding and origin.

Inoculation Method	Species	No. Coding	SPF Eggs	1-2-Day-Old Hatchlings	Breeder
Subcutaneous injection	Chickens	C 01 – C 08; (C 09 – C 12 *)	×		commercial (SPF)
	Ducks	D 01 – D 08; (D 09 – D 12 *)		×	regional/conventional
	Geese	G 01 – G 08; (G 09 – G 12 *)		×	regional/conventional
Mosquito bite	Geese	G 13 – G 16; (G 17 – G 20 ^†^)		×	

*, control groups; ^†^, geese not infected by mosquito bite.

**Table 2:** Vector competence of *Culex pipiens* biotype *pipiens* after intrathoracic injection, used to challenge domestic geese with WNV lineage 1.

	Infection	Dissemination	Transmission	
			WNV-Positive Heads	WNV-Positive Salivary Secretions
Efficiency (%) (95% CI)	92/92 (100) (96.1–100)	92/92 (100) (96.1–100)	92/92 (100) (96.1–100)	46/92 (50.0) (39.4–60.6)
Viral load (viral copies/µL of total RNA)	9.1 × 10^5^	2.9 × 10^4^	1.8 × 10^5^	NA

CI, confidence interval; NA, not applicable.

## Supplementary Materials

The following are available online at https://www.mdpi.com/2076-393X/8/3/507/s1, Table S1: Clinical scores of chickens (C), ducks (D), and geese (G), infected with WNV via subcutaneous injection (s.c.) or mosquito bite (m.b.), Table S2: Viremia (viral copies/µL of total RNA) in chickens (C), ducks (D), and geese (G) infected with WNV via needle injection (s.c.) or mosquito bite (m.b.) measured -2, 1-6, 10, 14, and 19/20/21 days post infection (dpi), Table S3: Antibody titers measured by VNT and a commercial ELISA of chickens (C), ducks (D), and geese (G) infected with WNV via needle injection (s.c.) or mosquito bite (m.b.) measured 10, 14, and 19/20/21 days post infection (dpi), Table S4: Viral load (viral copies/µL of total RNA) of organ samples of chickens (C), ducks (D), and geese (G) three weeks after infection with WNV via needle injection (s.c.) or mosquito bite (m.b.), Table S5: Histopathological lesions found in chickens and ducks infected subcutaneously with WNV and of the control birds (chickens and ducks), Table S6: Histopathological lesions of geese infected with WNV via needle injection (s.c.) or mosquito bite (m.b.) and of all control geese.
