# Supplementary material for: Pathogenicity of West Nile Virus Lineage 1 to German Poultry
Source: Vaccines (Basel). 2020 Sep 5;8(3):507. doi: 10.3390/vaccines8030507 (PMC7563189; doi:10.3390/vaccines8030507)
Supplement: Supplementary file 1 [file vaccines-08-00507-s001.pdf]

Supplementary Data

Table S1. Clinical scores of chickens (C), ducks (D), and geese (G), infected with WNV via subcutaneous injection (s.c.) or mosquito bite (m.b.). Clinical scores ranging from 0 to 4 are illustrated using a colour code (0 in white = healthy, 1 in yellow = mildly affected, 2 in orange = moderately affected, 3 in red = severely affected, and 4 in dark purple = death/euthanasia). Grey fields indicate the planned removal of birds 20 instead of 21 days post infection (dpi). All of the control birds (chickens, ducks, and geese) consistently had a clinical score of 0.

	Dpi / Bird	-2	1	2	3	4	5	6	7	8	9	10	11	12	13	14	15	16	17	18	19	20	21
S.c.	C 01	0	0	0	0	0	0	0	0	0	0	0	0	0	0	0	0	0	0	0	0	0	0
	C 02	0	0	0	0	0	0	0	0	0	0	0	0	0	0	0	0	0	0	0	0	0	0
	C 03	0	0	0	0	(1)	0	0	0	0	0	0	0	0	0	0	0	0	0	0	0	0	0
	C 04	0	0	0	0	0	0	0	0	0	0	0	0	0	0	0	0	0	0	0	0	0	0
	C 05	0	0	0	0	0	0	0	0	0	0	0	0	0	0	0	0	0	0	0	0	0	0
	C 06	0	0	0	0	0	0	0	0	0	0	0	0	0	0	0	0	0	0	0	0	0	0
	C 07	0	0	0	0	0	0	0	0	0	0	0	0	0	0	0	0	0	0	0	0	0	0
	C 08	0	0	0	0	0	0	0	0	0	0	0	0	0	0	0	0	0	0	0	0	0	0
	D 01	0	0	0	0	0	0	0	0	0	0	0	0	0	0	0	0	0	0	0	0	0	0
	D 02	0	0	0	0	0	0	0	0	0	0	0	0	0	0	0	0	0	0	0	0	0	0
	D 03	0	0	0	0	0	0	0	0	0	0	0	0	0	0	0	0	0	0	0	0	0	0
	D 04	0	0	0	0	0	0	0	0	0	0	0	0	0	0	0	0	0	0	0	0	0	0
	D 05	0	0	0	0	0	0	0	0	0	0	0	0	0	0	0	0	0	0	0	0	0	0
	D 06	0	0	0	0	0	0	0	0	0	0	0	0	0	0	0	0	0	0	0	0	0	0
	D 07 ‡	0	1	4	4	4	4	4	4	4	4	4	4	4	4	4	4	4	4	4	4	4	4
	D 08	0	0	0	0	0	0	0	0	0	0	0	0	0	0	0	0	0	0	0	0	0	0
	G 01	0	0	0	0	0	0	0	0	0	0	0	0	0	0	0	0	0	0	0	3	4	4
	G 02	0	0	0	0	0	0	0	0	0	0	0	0	0	0	0	0	0	0	0	0	0	0
	G 03	0	0	0	0	0	0	0	0	0	0	0	0	0	0	0	0	0	2	3	3	4	4
	G 04	0	0	0	0	0	0	0	0	0	0	0	0	0	0	0	0	0	0	0	0	0	0
	G 05	0	0	0	0	0	0	0	0	0	0	0	0	0	0	0	0	0	0	0	0	0	0
	G 06	0	0	0	0	0	0	0	0	0	0	0	0	0	0	0	0	0	0	0	0	0	0
	G 07	0	0	0	0	0	0	0	0	0	0	0	0	0	0	0	0	0	0	0	0	0	0
	G 08	0	0	0	0	0	0	0	0	0	0	0	0	0	0	0	0	0	0	0	0	0	0
M.b.	G 13	0	0	0	0	0	0	0	0	0	0	0	0	0	0	0	0	0	0	0	0	0	0
	G 14	0	0	0	0	0	0	0	0	0	0	0	0	0	0	0	0	0	0	0	0	0	0
	G 15	0	0	0	0	0	0	0	0	0	0	0	0	0	0	0	0	0	0	0	0	0	0
	G 16	0	0	0	0	0	0	0	0	0	0	0	0	0	0	0	0	0	0	0	0	0	
	G 17	0	0	0	0	0	0	0	0	0	0	0	0	0	0	0	0	0	0	0	0	0	
	G 18	0	0	0	0	0	0	0	0	0	0	0	0	0	0	0	0	0	0	0	0	0	0
	G 19	0	0	0	0	0	0	0	0	0	0	0	0	0	0	0	0	0	0	0	0	0	
	G 20	0	0	0	0	0	0	0	0	0	0	0	0	0	0	0	0	0	0	0	0	0	

‡, D 07 succumbed 2 dpi.

Table S2. Viremia (viral copies/µL of total RNA) in chickens (C), ducks (D), and geese (G) infected with WNV via needle injection (s.c.) or mosquito bite (m.b.) measured -2, 1-6, 10, 14, and 19/20/21

days post-infection (dpi). Samples of control birds (chickens, duck, and geese) were consistently negative.

	Dpi / Bird	-2	1	2	3	4	5	6	10	14	19/20/21
**S.c.**	**C 01**	Neg.	Neg.	NA	Neg.	NA	Neg.	Neg.	Neg.	Neg.	Neg.
	**C 02**	Neg.	Neg.	NA	Neg.	NA	Neg.	Neg.	Neg.	Neg.	Neg.
	**C 03**	Neg.	Neg.	NA	Neg.	NA	Neg.	Neg.	Neg.	Neg.	Neg.
	**C 04**	Neg.	Neg.	NA	Neg.	NA	Neg.	Neg.	Neg.	Neg.	Neg.
	**C 05**	Neg.	NA	1.3	NA	Neg.	NA	Neg.	Neg.	Neg.	Neg.
	**C 06**	Neg.	NA	1.2	NA	Neg.	NA	Neg.	Neg.	Neg.	Neg.
	**C 07**	Neg.	NA	0.5	NA	Neg.	NA	Neg.	Neg.	Neg.	Neg.
	**C 08**	Neg.	NA	0.4	NA	Neg.	NA	Neg.	Neg.	Neg.	Neg.
	**D 01**	Neg.	Neg.	NA	19.4	NA	Neg.	Neg.	Neg.	Neg.	Neg.
	**D 02**	Neg.	Neg.	NA	11.6	NA	Neg.	Neg.	Neg.	Neg.	Neg.
	**D 03**	Neg.	Neg.	NA	Neg.	NA	Neg.	Neg.	Neg.	Neg.	Neg.
	**D 04**	Neg.	Neg.	NA	4.6	NA	Neg.	Neg.	Neg.	Neg.	Neg.
	**D 05**	Neg.	NA	Neg.	NA	Neg.	NA	Neg.	Neg.	Neg.	Neg.
	**D 06**	Neg.	NA	Neg.	NA	Neg.	NA	Neg.	Neg.	Neg.	Neg.
	**D 07** ‡	Neg.	NA	NA	NA	NA	NA	NA	NA	NA	NA
	**D 08**	Neg.	NA	9.5	NA	Neg.	NA	Neg.	Neg.	Neg.	Neg.
	**G 01**	Neg.	Neg.	NA	29.5	Neg.	1.3	Neg.	Neg.	Neg.	Neg.
	**G 02**	Neg.	Neg.	NA	6.9	Neg.	Neg.	Neg.	Neg.	Neg.	Neg.
	**G 03**	Neg.	Neg.	NA	1.9	Neg.	Neg.	Neg.	Neg.	Neg.	Neg.
	**G 04**	Neg.	Neg.	NA	21.9	NA	Neg.	Neg.	Neg.	Neg.	Neg.
	**G 05**	Neg.	Neg.	Neg.	NA	Neg.	NA	Neg.	Neg.	Neg.	Neg.
	**G 06**	Neg.	NA	13.4	NA	7.9	NA	1.0	Neg.	Neg.	Neg.
	**G 07**	Neg.	NA	36.3	NA	7.6	NA	0.9	0.3	Neg.	Neg.
	**G 08**	Neg.	NA	8.6	NA	11.7	NA	Neg.	Neg.	Neg.	Neg.
**M.b.**	**G 13**	Neg.	Neg.	NA	56.1	NA	Neg.	NA	Neg.	Neg.	Neg.
	**G 14**	Neg.	NA	20.5	NA	Neg.	NA	Neg.	Neg.	Neg.	Neg.
	**G 15**	Neg.	NA	Neg.	NA	Neg.	NA	12.2	Neg.	Neg.	Neg.
	**G 16**	Neg.	NA	20.4	NA	13.2	NA	Neg.	Neg.	Neg.	Neg.
	**G 17**	Neg.	Neg.	Neg.	Neg.	Neg.	Neg.	Neg.	Neg.	Neg.	Neg.
	**G 18**	Neg.	Neg.	Neg.	Neg.	Neg.	Neg.	Neg.	Neg.	Neg.	Neg.
	**G 19**	Neg.	Neg.	Neg.	Neg.	Neg.	Neg.	Neg.	Neg.	Neg.	Neg.
	**G 20**	Neg.	Neg.	Neg.	Neg.	Neg.	Neg.	Neg.	Neg.	Neg.	Neg.

NA, not applicable; Neg., negative result (≤ 1 viral copy/5 µL of total RNA and cycle threshold ≥ 37); ‡, D 07 succumbed 2 dpi.

Table S3. Antibody titers measured by VNT and a commercial ELISA of chickens (C), ducks (D), and geese (G) infected with WNV via needle injection (s.c.) or mosquito bite (m.b.) measured 10, 14, and 19/20/21 days post infection (dpi). Samples taken before infection (-2 dpi) and those of the control birds (chickens, duck, and geese) were consistently negative (data not shown).

		10 dpi		14 dpi		19/20/21 dpi	
	**Bird**	**VNT titer (ND 50)**	**Signal/nose ratio (S/N %)**	**VNT titer (ND 50)**	**Signal/nose ratio (S/N %)**	**VNT titer (ND 50)**	**Signal/nose ratio (S/N %)**
**S.c.**	**C 01**	<10	35.9	40	44.9	45	25.7
	**C 02**	20	36.4	10	37.9	40	14.9
	**C 03**	<10	54.4	15	43.3	60	22.6
	**C 04**	<10	47.4	10	39.2	60	22.4
	**C 05**	10	36.1	30	34.6	60	19.5
	**C 06**	10	NA	<10	33.2	30	21.1
	**C 07**	<10	53.9	<10	42.9	40	23.8
	**C 08**	30	28.7	40	25.9	120	17.8
	**D 01**	80	17.5	320	13.7	480	6.2
	**D 02**	70	21.0	880	11.9	960	6.7
	**D 03**	40	15.2	80	12.4	320	6.6
	**D 04**	<10	26.4	60	15.9	640	6.6
	**D 05**	40	21.1	320	12.1	1920	7.0
	**D 06**	30	24.0	40	13.0	480	7.3
	**D 07** ‡	NA	NA	NA	NA	NA	NA
	**D 08**	30	20.3	40	11.0	80	8.5
	**G 01**	45	33.5	40	29.9	40	18.1
	**G 02**	10	32.8	10	27.9	40	15.6
	**G 03**	20	24.6	30	22.8	40	15.1
	**G 04**	30	24.1	10	27.7	60	16.1
	**G 05**	30	23.1	10	21.4	80	16.9
	**G 06**	25	29.6	10	25.7	30	16.1
	**G 07**	30	19.7	35	16.1	55	8.2
	**G 08**	10	22.7	<10	20.5	60	13.6
**M.b.**	**G 13**	15	40.3	20	39.5	40	20.1
	**G 14**	40	30.3	20	20.3	60	15.6
	**G 15**	60	55.3	20	35.9	80	29.0
	**G 16**	10	32.7	10	28.8	80	16.9
	**G 17**	<10	105.7	<10	102.2	<10	102.3
	**G 18**	<10	99.4	<10	99.7	<10	97.2
	**G 19**	<10	98.9	<10	100.6	<10	104.9
	**G 20**	<10	101.4	<10	101.4	<10	98.1

NA, not applicable; ‡, D 07 succumbed 2 dpi.

Table S4. Viral load (viral copies/µL of total RNA) of organ samples of chickens (C), ducks (D), and geese (G) three weeks after infection with WNV via needle injection (s.c.) or mosquito bite (m.b.). Samples of the control birds (chickens, ducks, and geese) were consistently negative (data not shown).

Bird	Viral load (copies/µL of total RNA)

		Brain	Liver	Spleen	Heart	Bursa cloacalis	Injection site
**S.c.**	**C 01**	Neg.	Neg.	Neg.	Neg.	Neg.	NA
	**C 02**	Neg.	Neg.	Neg.	Neg.	Neg.	NA
	**C 03**	Neg.	Neg.	Neg.	Neg.	Neg.	NA
	**C 04**	Neg.	Neg.	Neg.	Neg.	Neg.	NA
	**C 05**	Neg.	Neg.	Neg.	Neg.	Neg.	NA
	**C 06**	12.1	Neg.	Neg.	Neg.	Neg.	NA
	**C 07**	Neg.	Neg.	Neg.	Neg.	Neg.	NA
	**C 08**	Neg.	Neg.	Neg.	Neg.	Neg.	NA
	**D 01**	Neg.	Neg.	6.0	Neg.	Neg.	NA
	**D 02**	Neg.	Neg.	4.0	Neg.	Neg.	NA
	**D 03**	Neg.	Neg.	2.3	Neg.	Neg.	NA
	**D 04**	Neg.	Neg.	3.2	Neg.	Neg.	NA
	**D 05**	Neg.	Neg.	Neg.	Neg.	Neg.	NA
	**D 06**	1.3	Neg.	Neg.	Neg.	Neg.	NA
	**D 07** ‡	Neg.	1.4	17.2	3.6	Neg.	57.1
	**D 08**	Neg.	Neg.	0.4	Neg.	Neg.	NA
	**G 01**	2.7	Neg.	Neg.	Neg.	Neg.	NA
	**G 02**	1.1	Neg.	Neg.	Neg.	Neg.	NA
	**G 03**	0.2	Neg.	Neg.	Neg.	0.4	NA
	**G 04**	Neg.	Neg.	Neg.	Neg.	1.5	NA
	**G 05**	Neg.	Neg.	Neg.	Neg.	Neg.	NA
	**G 06**	8.1	Neg.	Neg.	Neg.	Neg.	NA
	**G 07**	2.0	Neg.	Neg.	Neg.	Neg.	NA
	**G 08**	0.5	Neg.	Neg.	Neg.	Neg.	NA
**M.b.**	**G 13**	Neg.	Neg.	Neg.	Neg.	Neg.	NA
	**G 14**	Neg.	Neg.	Neg.	Neg.	Neg.	NA
	**G 15**	Neg.	Neg.	Neg.	Neg.	Neg.	NA
	**G 16**	9.8	Neg.	Neg.	Neg.	Neg.	NA
	**G 17**	Neg.	Neg.	Neg.	Neg.	Neg.	NA
	**G 18**	Neg.	Neg.	Neg.	Neg.	Neg.	NA
	**G 19**	Neg.	Neg.	Neg.	Neg.	Neg.	NA
	**G 20**	Neg.	Neg.	Neg.	Neg.	Neg.	NA

NA, not applicable; Neg., negative result (≤ 1 viral copy/5 µL of total RNA and cycle threshold ≥ 37); ‡, D 07 succumbed 2 dpi.

Table S5. Histopathological lesions found in chickens and ducks infected subcutaneously with WNV and of the control birds (chickens and ducks). The table lists the most frequently affected organs separately, as well as additional findings.

Bird	Brain (encephalitis)	Heart (myocarditis)	Spleen	Additional findings
**WNV infected chickens**				
**C 01**	--	--	--	+ pericholangitis

**C 02**	--	+	--	+ pericholangitis
**C 03**	--	(+)	--	+ pericholangitis, bile duct proliferation
**C 04**	+(+) *	+	+ foll. hyp.	(+) pericholangitis
**C 05**	--	--	--	+ pericholangitis, bile duct proliferation
**C 06**	++ *	--	--	+ pericholangitis, bile duct proliferation
**C 07**	--	(+)	--	+ pericholangitis, bile duct proliferation
**C 08**	--	+	--	+ pericholangitis
**Control chickens**				
**C 09**	--	+	--	+ pericholangitis
**C 10**	--	--	--	+ pericholangitis, bile duct proliferation
**C 11**	--	--	--	+ pericholangitis
**C 12**	--	--	--	+ pericholangitis, bile duct proliferation
**WNV infected ducks**				
**D 01**	+ *	+(+)	--	+ pericholangitis, bile duct proliferation
**D 02**	+	++ *	--	++ pericholangitis, bile duct proliferation
**D 03**	(+)	(+)	--	--
**D 04**	(+)	+	+(+) foll. hyp.	--
**D 05**	+	--	--	--
**D 06**	+	(+) *	+ foll. hyp.	+(+) pericholangitis, bile duct proliferation
**D 07** §	--	--	++(+) *	++ diffuse lipidosis of the liver
**D 08**	--	+	--	+ pericholangitis, bile duct proliferation
**Control ducks**				
**D 09**	--	--	--	+ pericholangitis, + focal suppurative vasculitis
**D 10**	--	--	--	+ non-suppurative pancreatitis
**D 11**	--	--	--	--
**D 12**	--	--	--	--

(+), weak; +, mild; +(+), mild to moderate; ++, moderate; ++(+), moderate to severe; +++, severe; foll. hyp., follicular hyperplasia of the spleen; *, necrotizing inflammation; §, succumbed 2 dpi; pericholangitis/bile duct proliferation are most likely due to a mild parasitic infection, and the diffuse liver lipidosis might be a result of anorexia before death.

Table S6. Histopathological lesions of geese infected with WNV via needle injection (s.c.) or mosquito bite (m.b.) and of all control geese. The table lists the most frequently affected organs separately, as well as additional findings.

Bird	Brain (encephalitis)	Heart (myocarditis)	Spleen	Additional findings
**WNV-infected geese (s.c.)**				

**G 01**	(+)	--	++ *	++ multifocal acute subcutaneous hemorrhages<br>++ multifocal acute degenerative lipidosis with acute hemorrhages and acute non-suppurative hepatitis **
**G 02**	++	--	+ foll. hyp.	--
**G 03**	(+)	--	++ *	+ multifocal acute non-suppurative necrotizing hepatitis **
**G 04**	(+)	(+)	+ foll. hyp.	--
**G 05**	+	--	++ foll. hyp.	+ focal acute liver necrosis with hemorrhages and vacuolar degeneration, focal acute subcapsular hemorrhages
**G 06**	++ *	--	+ foll. hyp.	+ focal acute subcapsular hemorrhages in liver,<br>+ multifocal bile duct proliferation, + multifocal acute non-suppurative interstitial nephritis
**G 07**	+	--	--	+ focal acute hemorrhages with vacuolar degeneration and hepatocellular necrosis, + bile duct proliferation and multifocal subacute non-suppurative cholangitis
**G 08**	+	--	+ foll. hyp.	+ multifocal bile duct proliferation, + multifocal subacute non-suppurative cholangitis, multifocal acute subcapsular hemorrhages in the liver
**Control geese**				
**G 09**	--	--	--	+ focal acute subcapsular hemorrhages in the liver
**G 10**	--	--	--	+ oligofokal acute subcapsular hemorrhages in the liver
**G 11**	--	--	--	+ oligofocal acute subcapsular hemorrhages in the liver
**G 12**	--	--	--	(+) multifocal bile duct proliferation, + subacute multifocal non-suppurative cholangitis
**WNV infected geese (m.b.)**				
**G 13** ‡	+ *	--	+ foll. hyp.	+ oligofocal subacute non-suppurative fibronecrotizing arteritis in the spleen **
**G 14** ‡	+	(+)	+ *	--
**G 15** ‡	+(+)	+	++ foll. hyp.	--
**G 16** ‡	+	--	++ foll. hyp.	+ focal acute necrotizing enteritis with fibronecrotizing vasculitis **
**G 17**	--	--	--	--
**G 18**	--	--	--	--
**G 19**	--	--	--	--
**G 20**	--	--	--	--

(+), weak; +, mild; +(+), mild to moderate; ++, moderate; ++(+), moderate to severe; +++, severe; foll. hyp., follicular hyperplasia of the spleen; *, necrotizing inflammation; ‡, geese with seroconversion of those infected with WNV via mosquito bite; **, additional findings which might be directly associated with WNV infections, for example, lipidosis seen in G 01 can be explained by anorexia due to illness, and necrotizing alterations seen in G 03, G 13, and G 16 fit to a viral infection; subcapsular hemorrhages and vacuolar degeneration seen in several animals including the controls are consistent with the fatty liver hemorrhagic syndrome, and bile duct proliferations are frequently associated with mild subclinical endoparasitosis.
